# The rate of metabolism as a factor determining longevity of the *Saccharomyces cerevisiae* yeast

**DOI:** 10.1007/s11357-015-9868-8

**Published:** 2016-01-19

**Authors:** Mateusz Molon, Monika Szajwaj, Marek Tchorzewski, Andrzej Skoczowski, Ewa Niewiadomska, Renata Zadrag-Tecza

**Affiliations:** 1 Department of Biochemistry and Cell Biology, University of Rzeszow, Zelwerowicza 4, Rzeszow, 35-601 Poland; 2 Department of Molecular Biology, Maria Curie-Sklodowska University, Lublin, Poland; 3 Institute of Biology, Pedagogical University of Cracow, Krakow, Poland; 4 The Franciszek Górski Institute of Plant Physiology, Polish Academy of Sciences, Krakow, Poland

**Keywords:** Yeast, Longevity, Metabolism rate, RLS, Total lifespan, Cell energetic

## Abstract

Despite many controversies, the yeast *Saccharomyces cerevisiae* continues to be used as a model organism for the study of aging. Numerous theories and hypotheses have been created for several decades, yet basic mechanisms of aging have remained unclear. Therefore, the principal aim of this work is to propose a possible mechanism leading to increased longevity in yeast. In this paper, we suggest for the first time that there is a link between decreased metabolic activity, fertility and longevity expressed as time of life in yeast. Determination of reproductive potential and total lifespan with the use of *fob1Δ* and *sfp1Δ* mutants allows us to compare the “longevity” presented as the number of produced daughters with the longevity expressed as the time of life. The results of analyses presented in this paper suggest the need for a change in the definition of longevity of yeast by taking into consideration the time parameter. The mutants that have been described as “long-lived” in the literature, such as the *fob1Δ* mutant, have an increased reproductive potential but live no longer than their standard counterparts. On the other hand, the *sfp1Δ* mutant and the wild-type strain produce a similar number of daughter cells, but the former lives much longer. Our results demonstrate a correlation between the decreased efficiency of the translational apparatus and the longevity of the *sfp1Δ* mutant. We suggest that a possible factor regulating the lifespan is the rate of cell metabolism. To measure the basic metabolism of the yeast cells, we used the isothermal microcalorimetry method. In the case of *sfp1Δ*, the flow of energy, ATP concentration, polysome profile and translational fitness are significantly lower in comparison with the wild-type strain and the *fob1Δ* mutant.

**Electronic supplementary material:**

The online version of this article (doi:10.1007/s11357-015-9868-8) contains supplementary material, which is available to authorized users.

## Introduction

The yeast *Saccharomyces cerevisiae* is one of the model organisms used in research connected with aging processes. In 1959, Mortimer and Johnston observed that a single mother cell of the budding yeast *S. cerevisiae* has a limited reproductive ability (Mortimer and Johnston 1959). Gerontological studies have been dominated by a model of aging called “replicative aging”, which in its original version informs us about the number of daughter cells produced by an individual cell. It was supposed to be a model for research on aging of the cells capable of division in higher eukaryotes, including humans (Polymenis and Kennedy 2012).

A group of scientists led by Muller suggested that a limited reproductive potential of a cell may be connected with budding processes rather than aging (Muller et al. 1980). At the end of 1980s, it was postulated that accumulation of a “senescence factor” in mother yeast cells during subsequent cycles is the reason for limitation of the number of reproduction cycles. That factor is supposed not to be transported to the bud, or it can only be passed down to the bud in a small amount. That is why the daughters (virgin cells) maintain full reproductive potential irrespective of the reproductive age of the mother cell. One of the first candidates proposed as the senescence factor was extrachromosomal rDNA circles (Sinclair and Guarente 1997). The role of that factor was also assigned to the damaged mitochondria (Delaney et al. 2013; Lai et al. 2002), oxidatively damaged proteins (Aguilaniu et al. 2003) or thermal aggregates (Erjavec et al. 2007). A direct consequence of the choice of budding as a means of asexual reproduction of yeast cells is the asymmetric distribution of damaged macromolecules between the products of cytokinesis.

Reproductive potential can be regulated by knockout or overexpression of genes. A number of genes have been identified which, by being knocked out, can increase or decrease reproductive potential of cells (e.g. *FOB1*, *GPA2*, *HXK2* or *SOD1*, *RAD52*, respectively) (Kaeberlein and Kennedy 2005; Kaeberlein et al. 2005). Overexpression of genes can also increase the number of daughters produced by the mother cell (e.g. *RAS2*, *SIR2*) (Kaeberlein et al. 1999; Sun et al. 1994) or decrease fertility (e.g. *SNF1*) (Ashrafi et al. 2000). Moreover, reproductive potential can be regulated by environmental conditions, e.g. calorie restriction (Lin et al. 2004) or blocking ribosome biogenesis by treatment with diazaborine (Steffen et al. 2008).

An alternative explanation of the causes of limited proliferative capacity of yeast cells is the hypertrophy hypothesis (Bilinski and Bartosz 2006; Bilinski et al. 2012), which has already found strong experimental support (Wright et al. 2013; Yang et al. 2011; Zadrag-Tecza et al. 2009). According to that hypothesis, the factor responsible for the restriction of reproductive potential of yeast cells can be the critical size achieved by such cells, which makes them unable to continue their reproductive cycles. This shows that the rate of volume increase per generation determines the number of progeny. Continuous volume increase during each cell cycle is also an unavoidable consequence of the evolutionary choice of budding as a way of asexual reproduction.

Almost all laboratories in the world regard the number of generations that can be performed by a mother cell during its life as a unit of life expectancy. Despite the similarities in the shape of the survival curve of the different organisms, the unit of life expectancy for all organisms is always the time expressed in hours or years, except in the case of yeast, where the number of daughters (generations) is the measure (Sinclair et al. 1998). It has been long postulated that the number of generations is hardly a correct unit of lifespan as it informs us about cell fertility rather than lifespan (Gershon and Gershon 2000). In gerontology, time is the basic unit expressing life expectancy of a living organism; however, the commonly accepted way of presenting life expectancy of the *S. cerevisiae* yeast is totally different. Because of that, each factor or mutation that causes an increase in the reproductive potential is considered to have a life-prolonging role. There are facts which indicate that expressing yeast lifespan as a number of generations without taking into account the time parameter can alter dramatically the interpretation of the obtained results (Zadrag-Tecza et al. 2013). It was shown that the time of life of the studied mutants and their standard counterparts is relatively constant (differing by less than 30 %), irrespective of their genetic background. On the other hand, the value of replicative lifespan (number of daughters) can differ up to five times. This clearly shows that the applied units are absolutely not proportional, and hence drawing conclusions concerning longevity of yeast on such basis seems far-fetched (Zadrag-Tecza et al. 2013; Zadrag et al. 2008).

The idea that longevity of animals depends on the rate of metabolism has accompanied gerontology for many years. Generally, the rate of metabolism is expressed in units connected with oxygen consumption. Such approach can easily be applied primarily to homoeothermic organisms like mammals or birds. Steady-state energy consumed by these organisms is spent to a large extent on thermoregulation, movement and on functioning of the brain. In the case of unicellular and non-motile yeast, the energy is spent mainly on the synthesis of all cellular constituents, transport and intracellular movements. In yeast, energy expenditure on gene expression, especially translation seems to prevail as long as the main building blocks such as amino acids are available. Moreover, metabolism of the budding yeast is mainly fermentative, not oxidative, which makes measurement of oxygen consumption hardly applicable to the evaluation of the rate of metabolism. We have assumed that the most universal measure of metabolic rate will be the amount of calories produced by the cells living under optimum conditions. In that case, the optimum conditions mean growth on complete media on glucose, since under such conditions yeast cells reproduce at the highest rate.

Max Rubner, the creator of the rate of living theory, claimed that energy consumption by different species of mammals during their lives based on the unit of volume is similar despite differences in life expectancy (Rubner 1908). It was assumed that mammals possess a certain amount of energy to be used during their lifetime: they can use it at a quicker pace and live shorter or they can consume it economically, which allows them to live a longer life. That theory was extended in 1928 by R. Pearl (Pearl 1928). In this context, longevity is inversely proportional to the level of basic metabolism. This theory is connected not only with mammals but it is also supported by research done with the use of *Drosophila melanogaster*. As cold-blooded (poikilothermic) organisms, these insects live shorter in higher temperatures because the pace of their metabolism is quicker (Miquel et al. 1976). Our recent study showed that yeast also lived shorter in higher temperatures as opposed to the optimum temperature (Molon and Zadrag-Tecza 2015). However, explanation of the dependency between the growth rate of an organism and its life expectancy is still open for discussion.

The main aim of this work is to explain the mechanism that most likely leads to increased longevity in yeast. Here, we show the link between energy consumption, fertility and longevity in yeast. We also attempt at explaining the mechanism leading to longevity of yeast cells in the light of the rate of living theory of aging.

## Materials and methods

### Chemicals

Components of culture media were from BD Difco (Becton Dickinson and Company, Spark) except for glucose (POCh, Gliwice, Poland). [35S]-methionine was purchased from Hartmann analytic (Braunschweig, Niemcy). BacTiter-Glo™ Microbial Cell Viability Assay was purchased from Promega (Warszawa, Polska). FUN-1 was from Molecular Probes (Eugene, OR, USA). All other reagents, if not stated otherwise, were purchased from Sigma (Poznan, Poland).

### Yeast strains

Wild-type SP-4 (*MAT*α *leu1Δ arg4Δ*) (Bilinski et al. 1985) and isogenic mutants *fob1Δ* (*MAT*α *leu1Δ arg4Δ* YDR110W::kanMX4) (Zadrag-Tecza et al. 2013) and *sfp1Δ* (*MAT*α *leu1Δ arg4Δ YLR403W*::kanMX4) (for the purpose of this study).

Both mutants were obtained by Dr. Mateusz Molon. In order to perform gene disruption, the standard methods were used. Deletion cassette was amplified by PCR. DNA for the reaction was isolated from deletion mutants of the EUROSCARF collection (acc. no. Y04044 and Y05312).

For amplification, the following primers were used: kanMX4 (inside deletion cassette): GGATGTATGGGCTAA ATGTACG; sfp1_forward: CATATCGGTGCTTCTCTCTGG; sfp1_reverse: AGGAGAGACCAG ACAGAGCG; sfp1_verification (above to forward primer): CTCTTTTCTACCTGTCATCCC fob1_forward: GATCGAGGTTTCCAGGAAGAGC; fob1_reverse: GGAGCATTCCTCTGCATC TATTG; fob1_verification: CATCTTTTCATTGTACTCAGCGG. Mutants were selected onto yeast extract-peptone dextrose (YPD) medium containing geneticin (G-418) at the final concentration of 200 μg/ml. Deletion accuracy was checked by PCR using kanMX4 and verification primers.

### Media and growth conditions

Yeast was grown in a standard liquid YPD medium (1 % yeast extract, 1 % yeast Bacto-Peptone, 2 % glucose) or a yeast extract-peptone glycerol (YPG) medium (1 % yeast extract, 1 % yeast Bacto-Peptone, 2 % glycerol) on a rotary shaker at 150 rpm at a temperature of 28 °C.

### Kinetics of growth assay

Growth assays were carried out on liquid medium. Yeast cell suspension was incubated for 12 h in a shaking incubator at 28 °C (Heidolph Inkubator 1000 at 1200 rpm). The growth was monitored turbidimetrically in the Anthos 2010 type 17550 microplate reader at 600 nm by performing measurements at 1 h intervals for 12 h.

### Determination of reproductive potential, reproductive lifespan, post-reproductive lifespan and total lifespan

Yeast lifespan was determined with the use of the previously described method (Minois et al. 2005) with modification (Zadrag et al. 2008). Yeast cultures were grown in a rich YPD medium (1 % Bacto-Peptone, 1 % yeast extract, 2 % glucose, 2 % agar) to log phase. Ten microliter aliquots of each culture were dropped on separate YPD plates with solid medium containing Phloxine B (10 μg/ml). Forty single cells were micromanipulated for each experiment. The analysis was performed by micromanipulation using the Nikon Eclipse E200 optical microscope with an attached micromanipulator. The research was carried out for 16 h at 28 °C. After completion of the experiment for the day, the day dish was placed at +4 °C for 8 h. For each of the strains two to three biological repetitions were performed.

### Estimation of cell volume

Cell volume was estimated by analysis of microscopic images recorded during a routine procedure of determination of reproductive potential. Images were captured with a Nikon Eclipse E200 microscope equipped with the Olympus DP26 digital camera. Diameter of the cell was measured using the cellSens Standard software. Cell volume was calculated according to the method described by Zadrag-Tecza et al. (Zadrag-Tecza et al. 2009).

### Determination of the heat flow from the yeast population using isothermal calorimetry

Yeast cells were grown in YPG medium (1 % yeast extract, 1 % Bacto-Peptone, 2 % glucose) and at 28 °C until the exponential phase of growth. For the analysis, 5 × 10^7^ cells were used. Determination of heat emission was performed in the TAM III calorimeter. For the analysis, 20-min measurement was used. The results were analysed in the TAM Assistant software and expressed (in mJ) as an integral values of the area under the thermal power curves. For each of the strains, three independent biological repetitions were performed.

### Yeast polysome profile analysis

Yeast polysome profile was determined with the use of the previously described method (Warner et al. 1985). Yeast cells were cultured to OD_600_ 0.4–0.6 in YPD or appropriate minimal medium and treated with cycloheximide (Sigma-Aldrich) to the final concentration of 100 μg/ml for 20 min for preservation of polysomes. Afterwards, ice was added to the culture, and cells were harvested by centrifugation for 2 min at 8,000 rpm at 4 °C in a JLA-16.250 rotor (Beckman Coulter). Pellet of cells was resuspended in 10 ml of ice-cold lysis buffer [10 mM Tris-HCl pH 7.5, 100 mM NaCl, 30 mM MgCl_2_, 100 μg/ml cycloheximide, 1 mM PMSF, 6 mM β-Me, 1 nM pepstatin A, 10 nM leupeptin, 10 ng/ml aprotinin, 200 μg/ml heparin and RNase inhibitor (Sigma-Aldrich)], and cells were harvested by centrifugation for 3 min at 4500 rpm at 4 °C in a SX4250 rotor (Beckman Coulter). The cells were subsequently resuspended in 0.5 ml of lysis buffer and added to 0.5 ml of chilled acid-washed glass beads (diameter 425–600 μm, Sigma-Aldrich) and disrupted by vigorous shaking on a vortex mixer eight times for 45 s with 1 min cooling on ice. The cell lysate was clarified by centrifugation at 12,000 rpm for 10 min at 4 °C in a 12154-H rotor (Sigma-Aldrich), and 12 OD_260_ units of cell extract were loaded on a 7–47 % sucrose gradient [prepared in 50 mM Tris-acetate (pH 7.0), 12 mM MgCl_2_, 50 mM NH_4_Cl and 1 mM dithiothreitol] and centrifuged for 4.5 h at 26,500 rpm at 4 °C in a SW 32 Ti rotor (Beckman Coulter). Absorbance at 254 nm was detected along sucrose gradient using density gradient fractionation system (Brandel) and polysome profile was obtained.

### In vivo ^35^S-methionine incorporation

The ^35^S-radiolabelled-methionine incorporation test was performed as described previously (Carr-Schmid et al. 1999). Cells were grown to OD_600_ 0.5–0.7 in YPD, collected by centrifugation and subsequently re-suspended and grown for the next 15 min in minimal medium without methionine. Afterwards, the unlabelled methionine was added to the final concentration of 50 μM along with ^35^S-radiolabelled-methionine to 1 μCi/ml (31.75 TBq/mmol, 10 mCi/ml, Hartmann Analytic). At zero time and every 10 min, OD_600_ of the culture was determined and 1 ml cell culture was taken and added to 0.2 ml ice-cold 50 % TCA for protein precipitation. The samples were incubated on ice for 10 min, then at 80 °C for 20 min and filtered through the Whatman GF/C filters. The filters were washed with 5 % TCA, dried and submerged in scintillation liquid. Radioactivity was determined in a scintillator counter. Translational fitness of yeast strains was determined as ^35^S-methionine incorporation per OD_600_ per min.

### Measurement of ATP content

Measurement of ATP was performed using a BacTiter-Glo™ Microbial Cell Viability Assay Kit according to the manufacturer protocol (Promega). The yeast cells from the exponential phase culture were washed with sterile water and suspended to the final density of 10^6^ cells/ml in 100 mM phosphate buffer with pH 7.0 containing 0.1 % glucose and 1 mM sodium EDTA. A 100-μl cell suspension sample was used for determination purposes. Luminescence was recorded using the TECAN Infinite 200 microplate reader. The luminescent signal was proportional to the amount of ATP.

### Measurement of cell metabolic activity

Metabolic activity of yeast cells was assessed with FUN-1 according to the manufacturer protocol (Molecular Probes) with modification described by Kwolek-Mirek and Zadrag-Tecza (Kwolek-Mirek and Zadrag-Tecza 2014). The fluorescence of the cell suspension was measured after 15 min incubation in the dark and at 28 °C using the TECAN Infinite 200 microplate reader at *λ*_ex_ = 480 nm, *λ*_em_ = 500–650 nm. The metabolic activity of cells was expressed as a change in ratio of red (*λ* = 575 nm) to green fluorescence (*λ* = 535 nm). Cell observations were also carried out using a fluorescence microscope Olympus BX-51 equipped with a digital camera DP-72 and software cell^D.

### Measurement of superoxide anion generation

Generation of reactive oxygen species (superoxide anion) was assessed with dihydroethidine (DHET; final concentration 18.9 μM) (Benov et al. 1998). The yeast cells from the exponential phase culture were washed with sterile water and suspended to the final density of 10^8^ cells /ml in 100 mM phosphate buffer pH 7.0 containing 0.1 % glucose and 1 mM sodium EDTA. The kinetics of fluorescence increase due to oxidation of the dihydroethidine was measured using the TECAN Infinite 200 microplate reader at *λ*_ex_ = 518 nm and *λ*_em_ = 605 nm at the temperature of 28 °C.

### Determination of mitochondrial membrane potential

The mitochondrial membrane potential was assessed with rhodamine 123 according to the manufacturer’s protocol (Molecular Probes). The yeast cells from the exponential phase culture were washed with sterile water and suspended to the final density of 10^7^ cells /ml in 50 mM citrate buffer with pH 5.0, containing 2 % glucose. Rhodamine 123 was added to the final concentration of 5 μM. The fluorescence intensity was measured using the TECAN Infinite 200 microplate reader at *λ*_ex_ = 505 nm and *λ*_em_ = 534 nm.

The mitochondrial network was stained with rhodamine B hexyl ester—a red fluorescent stain which locates itself in mitochondria depending on mitochondrial membrane potential as described by Kwolek-Mirek et al. (Kwolek-Mirek et al. 2014). The mitochondrial network was visualised by fluorescence microscopy using the Olympus BX-51 microscope equipped with the DP-72 digital camera and cellSense Dimension software. The photos present the typical result of the duplicate experiment.

### Measurement of the oxygen consumption

The intensity or mitochondrial respiration was measured with oxygen electrode (Oxytherm, Hansatech, UK). 10^8^ cells were dissolved in 1 mL of fresh YP Glycerol medium and immediately loaded into the electrode chamber. The rate of oxygen consumption was followed in 5 min run at 28 °C. In order to verify whether the oxygen consumption was caused by mitochondrial respiration of yeast cells, sodium azide was added to the electrode chamber at the final concentration of 5 mM (in several control tests).

### Statistical analysis

Statistical analysis was performed with the use of StatSoft, Inc. (2011). [STATISTICA (data analysis software system), version 10. www.statsoft.com.] using the *t* test for independent samples in respect of the variable test and Dunnett’s post hoc test. The results were presented as mean ± standard deviation. Statistically significant differences were taken at *P* < 0.01.

## Results

**Fig. 1:**
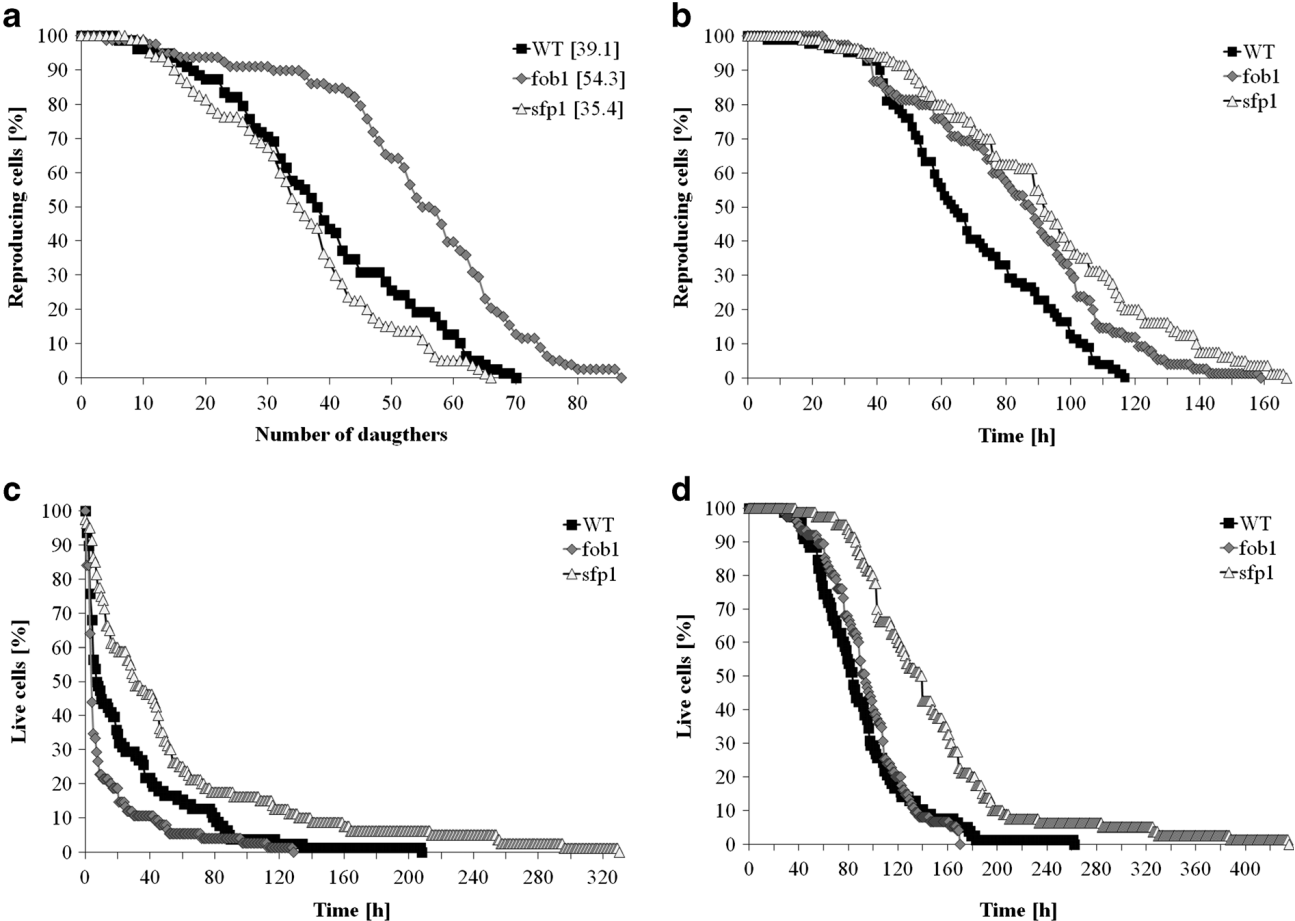
Comparison of the reproductive potential (**a**) reproductive lifespan (**b**), post-reproductive lifespan (**c**) and total lifespan (**d**) of the haploid wild-type yeast strain SP-4 and isogenic mutant strains *fob1Δ* and *sfp1Δ*. The mean value of the reproductive potential is shown in parentheses

For the purpose of the analysis, we used the *fob1*Δ and *sfp1*Δ mutants in the standard SP-4 genetic background. The *fob1*Δ mutant is regarded as a representative of long-lived mutants (if the number of daughters is taken as the measure of longevity instead of time units). In turn, the *sfp1*Δ mutant was initially described as short lived (Heeren et al. 2009); however, our results show that this is a long-lived mutant (if longevity is expressed in units of time) (Molon et al. 2015). In this paper, we first analysed the reproductive potential of these mutants in the background of the longest lived standard strain SP-4. As seen in Fig. 1a, the reproductive potential of the *fob1*Δ mutant is much extended, whereas that of the *sfp1*Δ mutant is slightly shorter than the reproductive potential of the wild-type strain. Reproductive lifespan (REPLS) of both mutants is noticeably extended (Fig. 1b). Conversely, post-reproductive lifespan (PRLS) of the *fob1*Δ mutant is strongly shortened, whereas that of the *sfp1*Δ mutant is substantially extended when compared to the wild-type strain (Fig. 1c). Consequently, the total lifespan TLS (sum of REPLS and PRLS) of the *fob1*Δ mutant, treated as a “longevity mutant”, does not differ from the total lifespan of the standard strain, in contrast to the *sfp1*Δ mutant which has a significantly increased TLS (Fig. 1d). These results confirm the previous findings that deletion of the *FOB1* gene does not result in longevity expressed in units of time, but only if lifespan is expressed as the number of daughters produced. This is a result of a negative correlation between PRLS and replicative lifespan (RLS). On the other hand, the *sfp1*Δ mutation increases not only REPLS but also PRLS, which results in a more than 50 % increase of TLS (Tab. 1). Based on these results, the *sfp1*Δ mutant can be reasonably described as the first sensu stricto longevity mutant of yeast.

**Fig. 2:**
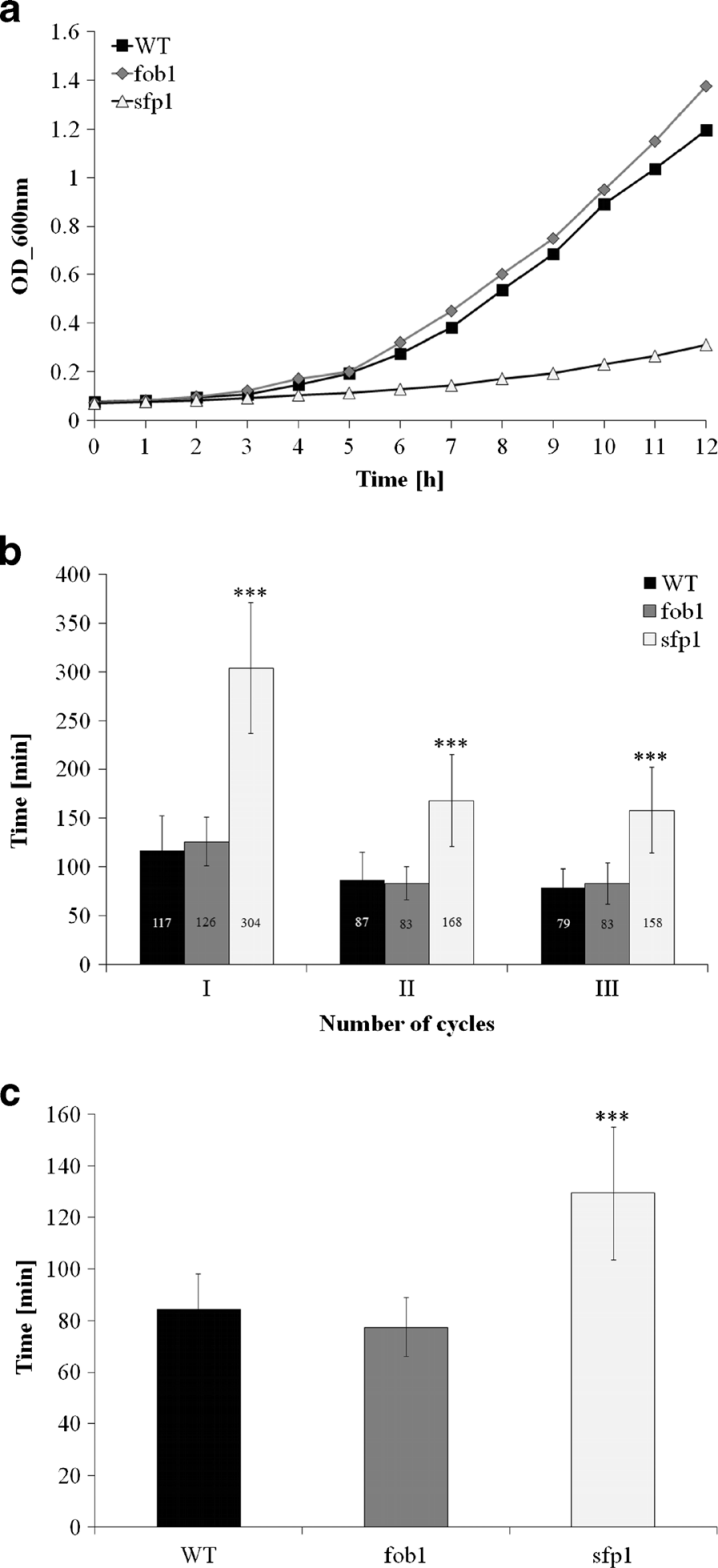
Comparison of growth kinetics (**a**), average doubling time of the first three generations (**b**), average doubling time during reproducing (**c**) of the haploid wild-type yeast strain SP-4 and isogenic mutant strains *fob1Δ* and *sfp1Δ*. ***P < 0.01 compared to the wild type strain

**Table 1:** The results represent the mean ± SD of the 80 cells analysed in two independent experiments (40 cells in each experiments). The statistical significance of differences in parameters examined between SP-4 (wild-type) and *fob1Δ*, *sfp1Δ* with the one-way analysis of variance (ANOVA)

Yeast strain	Reproductive potential (number of daughters)	Reproductive lifespan (hours)	Post-reproductive lifespan (hours)	Total lifespan (hours)
SP-4 (wild-type)	39.1 ± 15.5	67.6 ± 24.7	26.0 ± 36.2	90.1 ± 38.5
*fob1Δ*	54.3 ± 16.9***	82.8 ± 30.1***	13.5 ± 24.2	95.1 ± 30.8
*sfp1Δ*	35.36 ± 14.21***	92.2 ± 34.2***	54 ± 68.7***	146.22 ± 67.9***

***P < 0.001

**Fig. 3:**
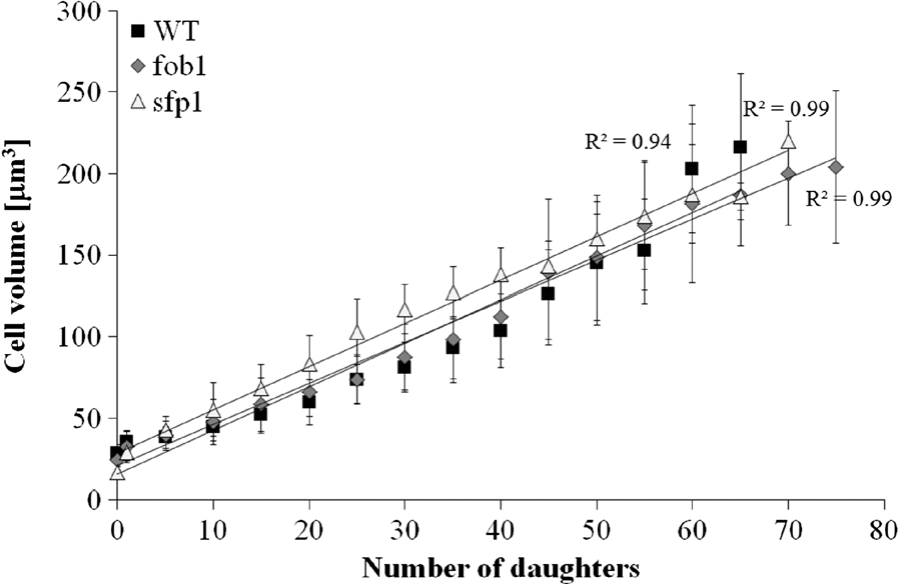
Dependence of cell volume on the number of daughters accomplished by mother yeast cells. Data are presented as mean (mean values were calculated from cells that performed a given number of reproductive cycles) ± SD. Data come from two independent experiments. The *bars* indicate SD

Next, we tested changes in cell volume during subsequent cycles of the studied strains. Our results indicate that the cells of the wild-type, *fob1*Δ and *sfp1*Δ strains achieved the same maximum volume when they stopped budding (Fig. 3). However, their growth rate per generation was different. In the case of *fob1*Δ, the cell volume increases more slowly per generation, and therefore cells of this strain perform more generations than the wild-type strain. On the other hand, *sfp1*Δ is characterised by long generation time, yet no significant differences in the volume growth per generation were observed compared to the wild-type strain. This strongly supported the basic assumptions of the hypertrophy hypothesis.

**Fig. 4:**
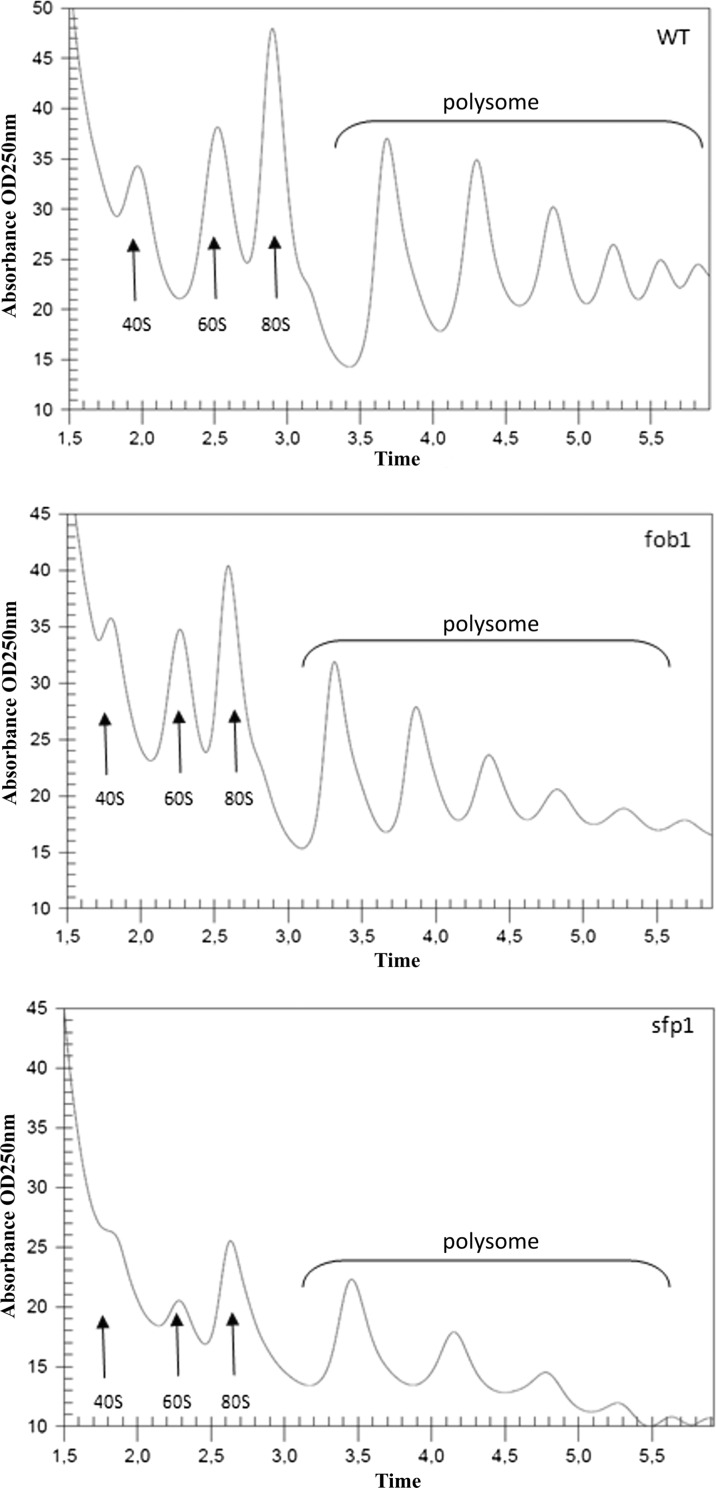
Polyribosome profiles of SP-4 wild-type, *fob1Δ* and *sfp1Δ* yeast strains. The *arrows* indicate positions of individual ribosomal subunits 40S and 60S, and monosomes (80S) and polysomes

**Fig. 5:**
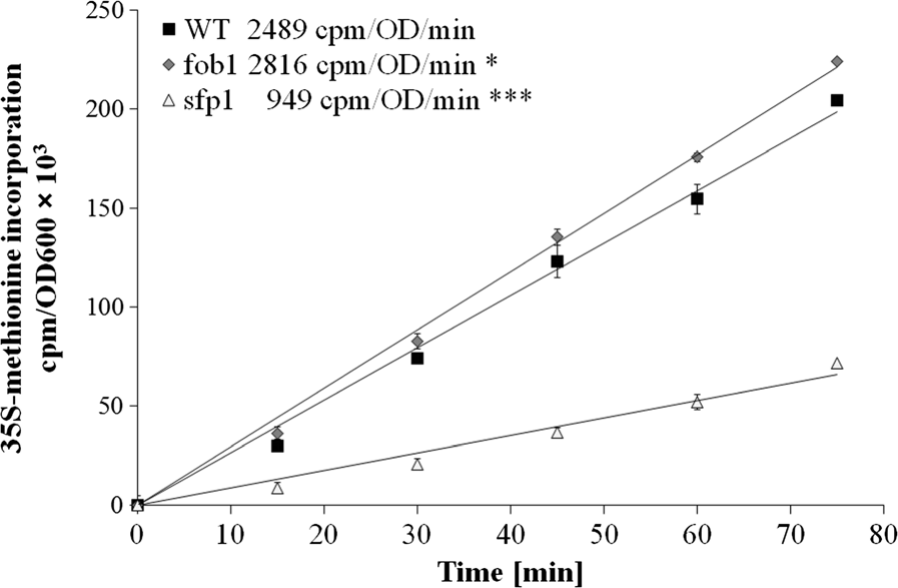
The rate of translation measure as in vivo ^35^S-methionine incorporation described as cpm/OD_600_. The results are presented as mean ± SD from three independent experiments. *P < 0.05 and ***P < 0.001 of significantly different values with respect to the wild-type strain estimated with ANOVA and Dunnet post-hoc test

**Fig. 6:**
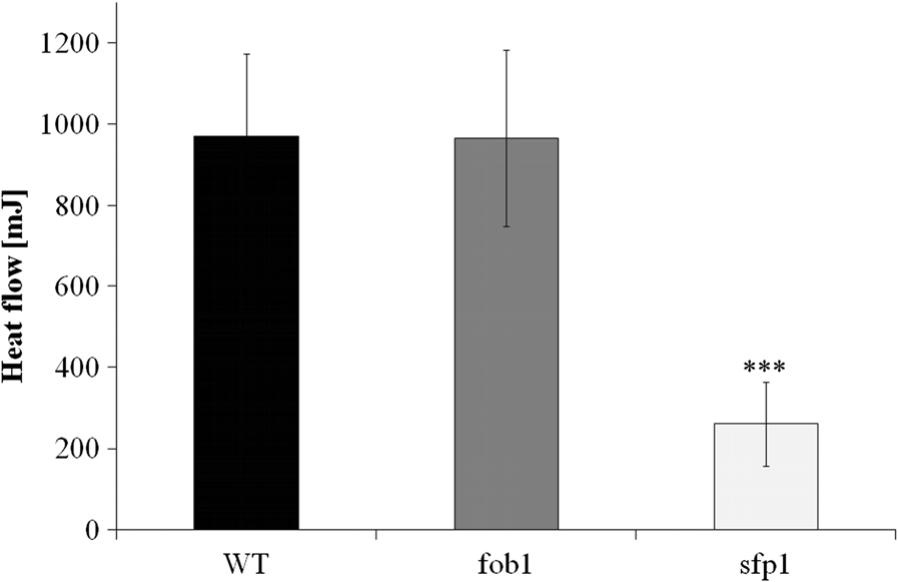
The flow of heat from the yeast population measured with isothermal calorimetry, defined as an area under the thermal power curve. Higher values of the heat flow indicate greater metabolic activity of the cells. The results are presented as mean ± SD from three independent experiments. ***P < 0.001 of significantly different values with respect to the wild-type strain estimated with ANOVA and Dunnet post-hoc test

**Fig. 7:**
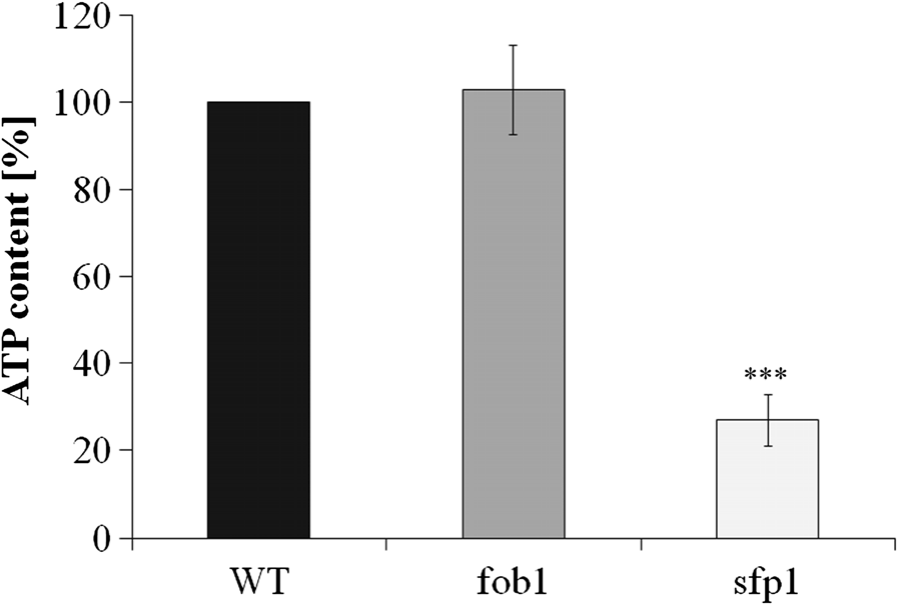
ATP content was determined using BactTiter-Glo™ Microbial Cell Viability Assay. Luminescence was recorded using the microplate reader. The luminescent signal was proportional to the amount of ATP. The results are presented as relative values (%) compared to the control for the wild-type strain. *Bars* indicate SD; *n* = 3; ***P < 0.001 of significantly different values with respect to the wild-type strain estimated with ANOVA and Dunnet post-hoc test

**Fig. 8:**
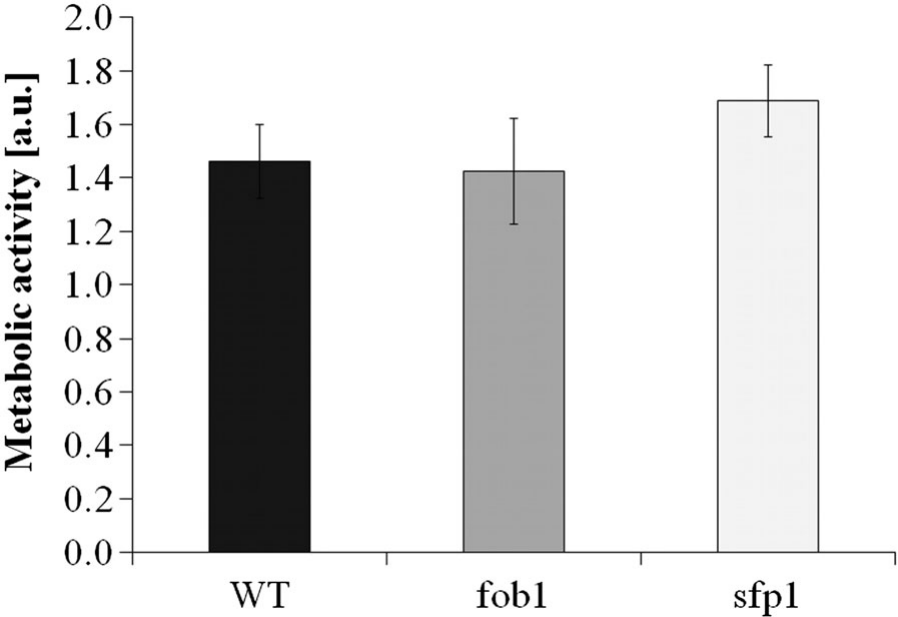
Metabolic activity of the cells (red/green ratio) was estimated with FUN-1 stain. Data are expressed as ratio of red (*λ* = 575 nm) to green (*λ* = 535 nm) fluorescence and presented as mean ± SD from three independent experiments. *Bars* indicate SD

Subsequently, we tested various physiological and cellular parameters to explain the reason for *sfp1*Δ cell longevity. A closer analysis of the generation time showed that the mutation in the *SFP1* gene decreases the rate of reproduction (Fig. 2a, c), mainly by extending the time of the first cell cycle of virgin cells, which dominate the population (Fig. 2b). “Virgin” in this case means cells that have not completed their first cell cycle. The difference between the wild-type and *fob1*Δ strains is very small (Fig. 2a–c). Then we looked for the factor that could trigger longevity in *sfp1*Δ mutant cells. Considering the kinetics of growth and generation time, it was reasonable to check the overall performance of the translational apparatus and metabolic activity of the tested strains. In view of the fact that the *SFP1* gene product is a transcription factor responsible for transcription of many genes involved in ribosome biogenesis, we first compared the profile of polysomes in the wild-type strain and the analysed mutants. The polysome profile analysis indicates that the *sfp1*Δ strain might have significant disorders in ribosome biogenesis and the formation of polysomes, indeed (Fig. 4). We observed a significant imbalance in the level of 40S and 60S subunits, especially low level of 60S was observed in comparison to 40S, which indicates disturbance in 60S biogenesis. Also, a low amount of 80S monosome and polysomes indicates that the translational machinery is impaired. Therefore, further analysis of the translational efficiency was performed using radioactively labelled methionine. The incorporation of [35S]-methionine enabled evaluation of so-called translational fitness, describing the overall performance of the translational apparatus. In the case of the *fob1*Δ strain, we observed a slight increase in that parameter, however following the main behaviour of wild-type strain. In contrast, the *sfp1*Δ mutant shows a significant reduction in the level of translational fitness (Fig. 5). The average level of methionine incorporation in this strain was approximately 60 % lower in comparison with the wild-type strain. Thus, the metabolic activity appears to be closely related to the efficiency of the translational apparatus, which is usually responsible for major energy consumption in yeast cells growing on glucose medium, which in turn might be visualised by the rate of reproduction. Therefore, we analysed the parameters useful for evaluating the metabolic/energetic status of the cells. The *S. cerevisiae* cells are non-motile and spend energy only on intracellular metabolic events. It therefore seems reasonable to assume that heat production through the population of yeast cells can become a determinant of the rate of metabolism connected primarily with cell reproduction. Measurements of heat flow from a population of the wild-type strain and the two mutants in question showed significant differences in the amount of the emitted heat only in the case of the *sfp1*Δ mutant (Fig. 6). The average amount of heat flow for that strain corresponds to approximately 27 % of the heat flow of the wild-type strain. Next, we determined the intracellular ATP levels. As in the case of heat emission, the *SFP1* mutant differs significantly from the other two strains also in terms of ATP content. The amount of ATP in *sfp1*Δ is 25 % of the ATP quantity of the wild-type strain (Fig. 7). There are no statistically significant differences between the *fob1*Δ and wild-type strains. Given these significant differences in the amount of heat emitted and the ATP content between the wild-type and *sfp1Δ* strains, we also checked cell vitality with the use of the yeast-specific fluorescent marker FUN-1. The dye as a membrane-permeant non-fluorescent precursor requires a conversion by the intracellular enzymes to a fluorescent product. The results indicate that despite numerous disorders related to ribosome biosynthesis, translation, and reduction in the level of cellular ATP content, the *sfp1Δ* mutant does not show differences in cell vitality (understood as the ability to perform basic metabolic processes) compared to the wild-type strain or *fob1Δ* (Fig. 8).

**Fig. 9:**
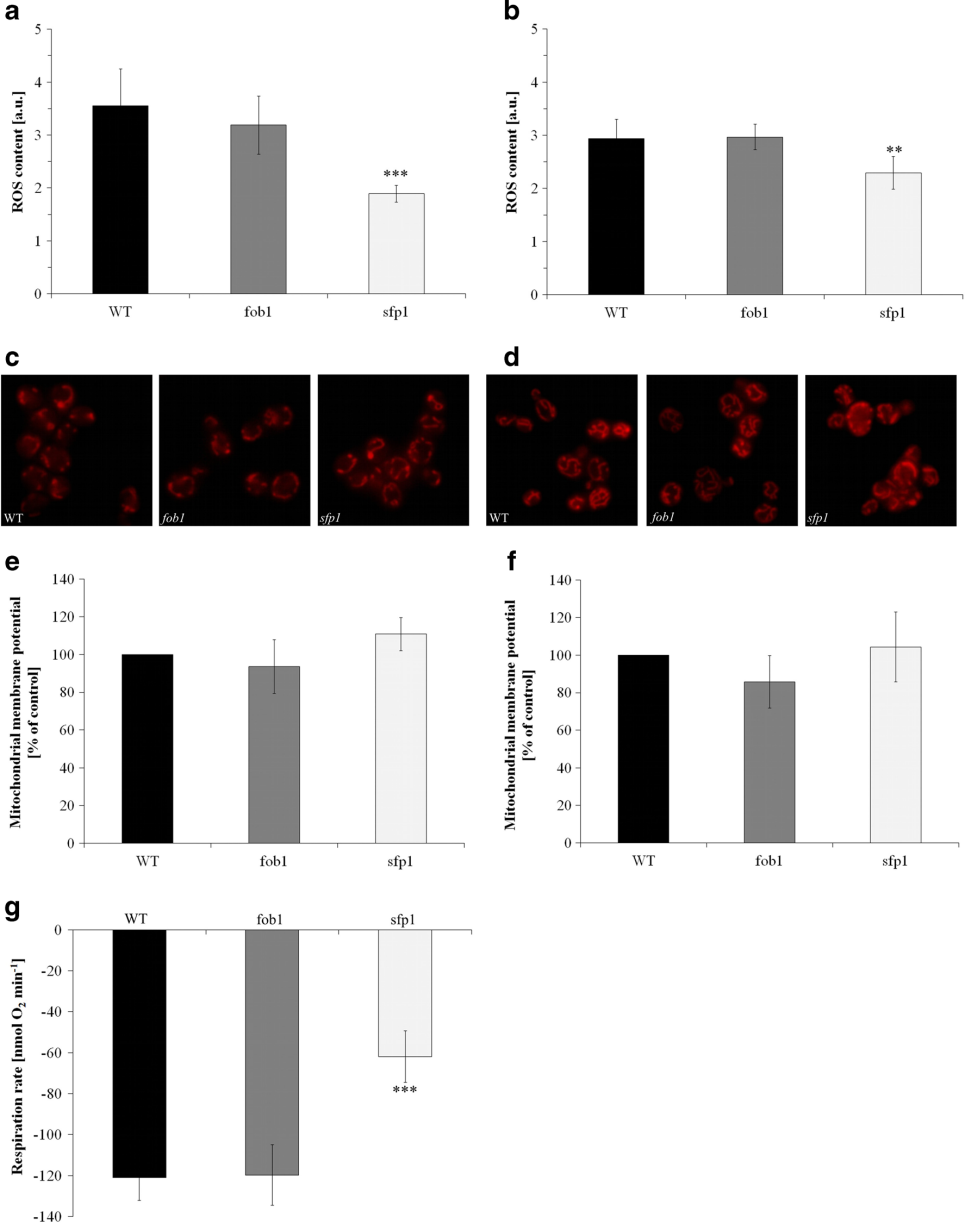
Superoxide anion (one type of ROS) content was estimated with the fluorescent probe dihydroethidine for yeast cells grown on liquid medium with glucose (**a**) or with glycerol (**b**). Data are presented as mean ± SD from at least three independent experiments. *Bars* indicate SD; **P < 0.01 and ***P < 0.001 of significantly different values with respect to the wild-type strain estimated with ANOVA and Dunnet post-hoc test. The mitochondrial network was stained with rhodamine B hexyl ester—a red fluorescent stain. The photos present the typical result of the duplicate experiment for yeast cells grown on medium with glucose (**c**) or glycerol (**d**). The mitochondrial membrane potential was assessed with rhodamine 123 for yeast cells grown on medium with glucose (**e**) or glycerol (**f**). Data come from at least three independent experiments and are presented as per cent of control (wild-type strain). *Bars* indicate SD; **P < 0.01 and ***P < 0.001 of significantly different values with respect to the wild-type strain estimated with ANOVA and Dunnet post-hoc test. The oxygen consumption was followed by oxygen electrode (Oxytherm, Hansatech, UK) for 5 min at 28 °C for yeast cells grown on medium with glycerol (**g**). Data are expressed as respiration rate [nmol O_2_ min^−1^] and presented as mean ± SD from three independent experiments. *Bars* indicate SD; **P < 0.01 and ***P < 0.001 of significantly different values with respect to the wild-type strain estimated with ANOVA and Dunnet post-hoc test

We also tested the parameters related to the functional state of mitochondria as structures responsible for the energy potential of the cells. The analysis of the mitochondrial membrane potential showed no statistically significant differences between the analysed yeast strains (Fig. 9e, f) both in the case of cells grown on medium with glucose where the fermentation metabolism prevails (Fig. 9e) or glycerol where the cells can only use the aerobic metabolism (Fig. 9f). This also confirms the image of the mitochondrial network where the fluorescent dye was used, the accumulation of which in mitochondria depends on mitochondrial membrane potential (Fig. 9c, d). There are clear variations in the degree of the mitochondrial network development depending on the carbon source in the medium. In the case of a medium containing glucose, single mitochondria are observed (Fig. 9c), while in the case of medium containing glycerol, the mitochondrial network is much more developed (Fig. 9d). However, no differences were observed between the analysed yeast strains. The observed oxygen consumption rates indicate that there are no statistically significant differences between the *fob1Δ* and wild-type strains while the *sfp1Δ* mutant shows a statistically significantly lower value of this parameter (Fig 9g). The suppression of O_2_ uptake in the presence of sodium azide (Fig. S1, supplementary data) indicates an inhibition of cytochrome-c oxidase activity. Considering that mitochondria are the main endogenous sources of reactive oxygen species (ROS), we performed the determination of the ROS level. The results of ROS content (represented by superoxide anion) indicate that there are no statistically significant differences between the *fob1Δ* and wild-type strains. On the other hand, a lower value of this parameter is shown by the *sfp1Δ* mutant. A similar relationship was observed both on media containing glucose (Fig. 9a) and on media containing glycerol (Fig. 9b).

Interestingly enough, in almost all of these assays, the *fob1Δ* mutant was always at a level close to the reference strain, and the differences concerned the long-lived *sfp1Δ* mutant only.

## Discussion

In all of these studies, selection of the appropriate unit was crucial to allow for correct interpretation of the results. In the studies of yeast cell aging, the most frequently used unit of age was the value of RLS expressed as the number of daughters. The results of our recent studies where mutants with increased reproductive potential were used strongly suggested that longevity of all organisms should be expressed in units of time because it allows one to draw dependable conclusions and make a comparison between species possible (Molon et al. 2015).

For the analysis, we used two mutants, namely *fob1Δ* and *sfp1Δ* in SP-4 genetic background. Compared to other strains, the SP-4 wild-type strain used in our study has the highest reproductive potential among the standard strains used, although it was never selected for that variable. It is commonly known that the phenotypic effects of the mutations are often strongly influenced by genetic background. Our results and the data from other papers on the same gene can sometimes bring divergent results, thereby hindering their interpretation. The *fob1Δ* mutant is the most frequently presented mutant in the literature and is defined as “long-lived” (in terms of increase of reproductive potential as compared to the wild-type strain) (Defossez et al. 1999; Kaeberlein et al. 1999; Lin et al. 2003)*.* In turn, *SFP1* deletion may have quite a different effect on reproductive potential (Heeren et al. 2009; Molon et al. 2015); however, it causes a statistically significant increase in total lifespan (Molon et al. 2015). Furthermore, according to our observations, *sfp1Δ* is the first longevity strain in terms of time of life; the question was now why that particular strain lives longer. Our studies demonstrate that deletion of the *SFP1* gene causes a slight decrease in the reproductive potential. On the other hand, the reproductive potential is significantly extended in *fob1Δ* yeast strain, which is consistent with the previous data (Defossez et al. 1999; Kaeberlein et al. 1999; Lin et al. 2003; Molon et al. 2015). In the case of *sfp1Δ*, a decrease in the number of daughters produced by cells is consistent with the data obtained by Heeren et al. (Heeren et al. 2009), but are not consistent with the recent data (Molon et al. 2015). It seems that the quantitative effect of this mutation is strain dependent.

To explain the differences in the reproductive potential, we considered the hypertrophy hypothesis (Bilinski and Bartosz 2006; Bilinski et al. 2012), which had already been widely discussed (Bilinski 2012; Ganley et al. 2012; Kaeberlein 2012; Wright et al. 2013). These results (Fig. 2) indicate that the rate of volume growth per generation is an important factor in achieving high fertility by a yeast cell. However, even though that relation can explain changes in reproductive potential, it cannot explain longevity expressed in time units since it represents only a certain part of the whole life of yeast cells. Reproductive lifespan is extended in both analysed mutants when we compare it with the wild-type strain. The *fob1Δ* mutation significantly increases reproductive potential, but in the case of *sfp1Δ*, the significant increase is mainly related to the doubling time and PRLS (Figs. 1b and 3c). After the last cell cycle, the *sfp1Δ* cells do not die so early, drastically exceeding time for wild-type and *fob1Δ* strains. We suppose that the very short post-reproductive lifespan in *fob1Δ* (Fig. 1c) may result from utilisation of some important resources necessary for reproduction. Significant extension of that time in *sfp1Δ* yeast strain is probably caused by slow energy utilisation, supporting low-energy consumption hypothesis in longevity extension. Our previous data indicated that an increase in the number of daughters produced by a cell shortens the time of life of the cell after reproduction (Molon et al. 2015; Zadrag-Tecza et al. 2013). This can be connected with significant energy load for the mother cell resulting from production of daughters and the increase in the cell volume during subsequent cycles and reproduction. By totalling the values of reproductive and post-reproductive lifetime, we arrive at the value of the total lifespan (TLS), which was presented for the first time in 2008 (Zadrag et al. 2008). The parameter can provide a basis for determining whether or not, we deal with a sensu stricto longevity phenotype. It seems that certain mutations can modulate duration of those two phases in a different way; therefore, their analyses are necessary for drawing firm conclusions about longevity phenotype of yeast strain (Molon et al. 2015; Zadrag-Tecza et al. 2013). Mutation in the *FOB1* gene does not influence the total lifespan, which is consistent with our previous observations (Molon et al. 2015; Zadrag-Tecza et al. 2013). The *sfp1Δ* mutation, on the other hand, significantly increases the total lifespan irrespective of the genetic background (this study; (Molon et al. 2015)). The common feature of the *SFP1* gene deletion regardless of the genetic background is that it decreases the rate of cell growth in the population and causes statistically significant prolongation of the average doubling time (Blumberg and Silver 1991). A very slow kinetics of the population growth as well as long generation time indicates potential problems, especially in biosynthetic efficiency of cells, which is usually responsible for significant energy resources utilisation. Thus, an attempt to explain mechanisms responsible for yeast longevity was the analysis of parameters connected with cell bioenergetics. A first parameter to define biosynthetic efficiency of cells is the polysome profile analysis, reflecting metabolic state of translational apparatus. The analysis of this parameter confirmed disorder in ribosome biogenesis and decrease in the level of the whole polysome profile for the *sfp1Δ* mutant (Fig. 4). The fitness of translational apparatus was further investigated by the means of the level of [35S]-methionine incorporation, which brings information about the biosynthetic capacity of translational apparatus (Fig. 5). The level of protein biosynthesis as far as the long-lived *sfp1Δ* mutant is concerned dropped by about 60 %, once again confirming significant dysfunction of translational apparatus, supporting previous observation, where *sfp1Δ* mutant was associated with global translation impairment (Fingerman et al. 2003; Thorburn et al. 2013). As far as mutation in the *FOB1* gene is concerned, no significant changes were observed. It is worth indicating that ribosome biogenesis is one of the most energy-consuming cell processes. Under optimal conditions more than 50 % of transcripts, which originate from RNA II polymerases, are connected with ribosomal proteins or with other trans-acting factors involved in ribosome biogenesis (Martin et al. 2006; Rudra and Warner 2004; Warner 1999). The level of translation can directly regulate a number of physiological parameters of cells; consequently, there has been a need for analysis of metabolic activity of cells in the tested strains. Measurement of the metabolic rate with the use of the isothermal microcalorimetry technique can give a meaningful result (Criddle and Hansen 1999). The produced heat is considered to be the by-product of all metabolic processes (Criddle et al. 1991). Hence, the thermal energy emitted outside the cell comes from the basic metabolism. Our studies showed that the *sfp1Δ* strain emits very little thermal energy; therefore, the population of this strain shows a very low “metabolic activity” in comparison with the wild-type strain and *fob1Δ* mutant (Fig. 6). Similar conclusion can be drawn from the analysis of the ATP level, which indicates that the significant decrease of the ATP content in the long-lived *sfp1Δ* mutant (Fig. 7) can be correlated with the demand for energy. This could mean that a cell adjusts the concentration of ATP to the current metabolic status or demand for that molecule. This is consistent with the opinion that synthesis of ribosomes is one of the most energy-demanding processes performed by the cell (Warner 1999). The results of analyses showing cell vitality performed with the use of the fluorescent probe FUN-1 indicate that the cells of the *sfp1Δ* strain, despite considerably lower protein biosynthesis, are metabolically efficient and able to perform basic metabolic processes, with metabolic activity similar (or even slightly higher) to the wild-type strain and *fob1Δ* mutant. These results show that the cells are metabolically efficient and capable of normal functioning. In this case, the mutation leads to slowing down cell growth and rate of reproduction without impairing general vitality of the cells. Therefore, it seems reasonable to postulate that a mutation in the *SFP1* gene does not affect the metabolic possibilities of the yeast cell. We expected that as a result of the removal of the RNA polymerase II transcription factor (Sfp1p), the cells would adjust the ATP amount to their needs, but its lowered level still ensures the basic level of cell metabolism. Moreover, the results showing the level of superoxide anion production, mitochondrial membrane potential or oxygen consumption rate, which reflect the functional state of mitochondria (Fig. 9a–f), indicate that the consequences of the lack of Sfp1p, such as low levels of ATP and heat emissions, do not result from the distorted structure or function of the mitochondria. The hypothesis of ATP level adjustment to the current needs of the cell seems more likely in this case. The low level of ATP in the case of the *sfpΔ* mutant seems to be the effect of the reduced demand resulting from the disorders of the most energy-intensive process such as protein biosynthesis, rather than the cause of reduction in metabolic efficiency of cells and thereby of the slowing down of growth, prolongation of generation time and other processes.

Taking into consideration the rate of living theory (Pearl 1928), the presented results may lead to the conclusion that cell bioenergetics can be responsible for the *sfp1Δ* mutant’s longevity. The extended time of life of the *sfp1Δ* mutant can also result from the secondary effects of lowering the rate of building of cell translational potential. In most cases, the mutations that extend the generation time (usually by increasing the G1 phase of the cell cycle) cause an increase in the growth rate of cell volume per generation, which results in lowering the value of RLS. In the *sfp1Δ* mutant, the rate remains unchanged, whereas the generation time strongly increases. Inverse relationship of the RLS and PRLS values strongly suggests that cells which invest more in progeny become easily depleted by some crucial molecules than those producing less daughters.

We are, however, aware of the fact that comparing such evolutionarily distant species or drawing straightforward conclusions from the behaviour of mammals or phenomena observed in yeast is bound to be risky. First of all, the units of assessment of the rate of metabolism are unavoidably different because of too large differences in overall metabolism. Furthermore, in mammals, we measure the resting metabolism—a notion that does not apply to free living unicellular organisms. In contrast to animals, yeast cells are never “resting”. Their life strategy is to invest in reproduction when resources are available or to prepare themselves for the starvation stress when their level diminishes.

## Electronic supplementary materials

Below is the link to the electronic supplementary material.

ESM 1 Figure S1. Inhibition of cytochrome-c oxidase activity by adding sodium azide to a final concentration of 5 mM. (JPG 417 kb)
