# Supplementary figures and images for: The rate of metabolism as a factor determining longevity of the Saccharomyces cerevisiae yeast
Source: Age (Dordr). 2016 Jan 19;38(1):11. doi: 10.1007/s11357-015-9868-8 (PMC5005888; doi:10.1007/s11357-015-9868-8)

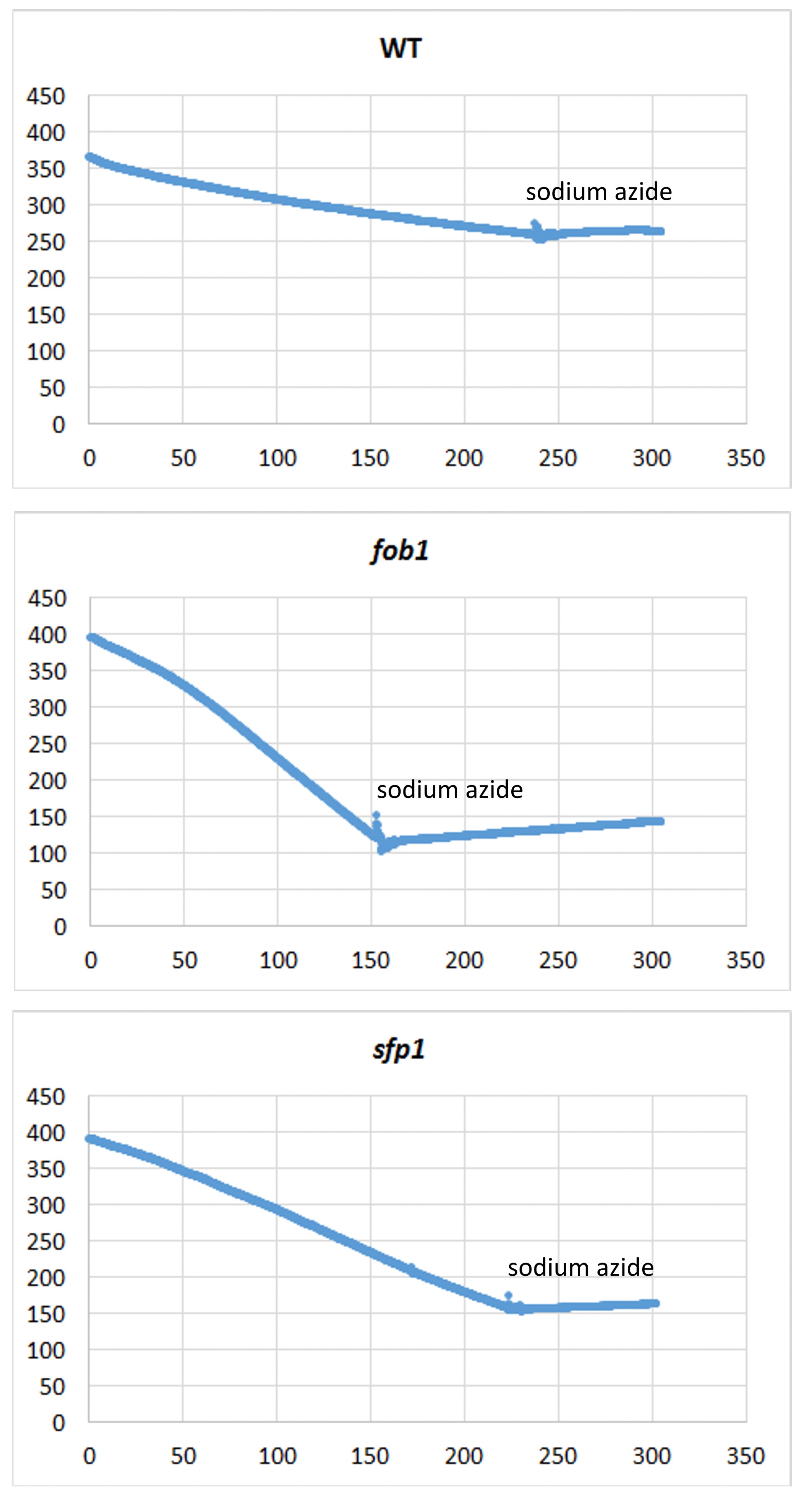

Supplement: Supplementary file 1 — Figure S1. Inhibition of cytochrome-c oxidase activity by adding sodium azide to a final concentration of 5 mM. (JPG 417 kb) [file 11357_2015_9868_MOESM1_ESM.jpg]
